# Design and other methodological considerations for the construction of human fetal and neonatal size and growth charts

**DOI:** 10.1002/sim.8000

**Published:** 2018-10-23

**Authors:** Eric O. Ohuma, Douglas G. Altman, for the International Fetal and Newborn Growth Consortium for the 21st Century (INTERGROWTH-21st Project)

**Affiliations:** ^1^ Nuffield Department of Women's & Reproductive Health University of Oxford, John Radcliffe Hospital Headington, Oxford, OX3 9DU UK; ^2^ Centre for Statistics in Medicine, Botnar Research Centre, Nuffield Department of Orthopaedics, Rheumatology and Musculoskeletal Sciences University of Oxford Windmill Road, Oxford, OX3 7LD UK

**Keywords:** design, fetal, growth charts, methodological considerations, neonatal

## Abstract

This paper discusses the features of study design and methodological considerations for constructing reference centile charts for attained size, growth, and velocity charts with a focus on human growth charts used during pregnancy. Recent systematic reviews of pregnancy dating, fetal size, and newborn size charts showed that many studies aimed at constructing charts are still conducted poorly. Important design features such as inclusion and exclusion criteria, ultrasound quality control measures, sample size determination, anthropometric evaluation, gestational age estimation, assessment of outliers, and chart presentation are seldom well addressed, considered, or reported. Many of these charts are in clinical use today and directly affect the identification of at‐risk newborns that require treatment and nutritional strategies.

This paper therefore reiterates some of the concepts previously identified as important for growth studies, focusing on considerations and concepts related to study design, sample size, and methodological considerations with an aim of obtaining valid reference or standard centile charts. We discuss some of the key issues and provide more details and practical examples based on our experiences from the INTERGROWTH‐21^st^ Project. We discuss the statistical methodology and analyses for cross‐sectional studies and longitudinal studies in a separate article in this issue.

## 1 INTRODUCTION

The earliest and most famous record of human growth is of the height of one boy, measured nearly every 6 months from birth to 18 years. It was made during the years 1759–1777 by Count Philibert Gueneau de Montbeillard using his son and later published by Buffon in a supplement to the *Histoire Naturelle*.^1^ Since then, reference charts have become the norm as a tool for monitoring human growth.

A reference chart depicts a family of curves representing a few selected centiles of the distribution of some physical characteristic of the reference population as a function of age. Such charts allow an individual to be placed in the context of like individuals. Charts of measurements are useful for assessing humans at all stages: fetuses, neonates, children, and adults. Our main focus here is on fetal growth up to newborn size at birth, but most of the concepts are relevant for child growth too. These concepts are also relevant in areas such as clinical chemistry that deals with the development of reference values for biochemical quantities, eg, albumin, creatinine, ferritin, and thyroid‐stimulating hormone, during pregnancy periods.^2^, ^3^, ^4^

Growth charts are intended to aid clinical judgments. Fetal growth charts are primarily used to compare the size of a fetus with reference data when gestational age (GA) is known at a specified time,^5^ to estimate GA from fetal size (eg, crown‐rump length, biparietal diameter, and fetal head circumference are commonly used for this purpose),^6^, ^7^, ^8^ and to assess a fetus's rate of growth between two time points (velocity).^9^, ^10^

Charts of size at birth are used as a screening tool in the identification or classification of newborn size as small, appropriate, or large for a specified GA at birth, based on cut‐off points on a specified reference chart.^11^, ^12^ Centiles are often used to help clinical decisions, for example, a fetus classified as being >97^th^ centile according to an estimated fetal weight chart would prompt clinicians to either deliver early or consider a cesarean section to avoid complications that may be associated with delivering a large baby. Many research applications use charts to describe the average pattern. However, reference charts are used clinically to identify individuals whose measurements fall beyond extreme centiles (for example, below the 3^rd^, 5^th^, or 10^th^ centiles or above the 90^th^, 95^th^, or 97^th^ centiles). These extreme centiles are likely to be indicative of growth restriction or other clinical complications affecting growth.

In most fields of medicine that require the identification of what is regarded as *normal*, internationally accepted classifications, cut offs, or standards are applied. These global cut offs are chosen as a result of evidence of adverse outcomes in relation to the target measurement, such as in the definitions of hypertension, anemia, and diabetes. In fetal medicine, however, no such standard values exist. The INTERGROWTH‐21^st^ systematic reviews of published reference charts of fetal biometry, pregnancy dating, and neonatal size^13^, ^14^, ^15^, ^16^ revealed wide variations between the centile values reported by published studies. There was considerable methodological heterogeneity: The charts were based on different populations and created with different sample selection, methodology, and statistical modeling methods.^13^, ^17^ Of all the many types of chart in use around the world, reference charts are one of the most widely used—yet few methodologists and statisticians are working to improve the methodology in this area.

The shortcomings of existing studies, as revealed by the INTERGROWTH‐21^st^ systematic reviews, are the motivation for discussing key issues in study design that underpin how to plan a quality study.

The key methodological considerations for the good design of studies to develop reference charts include, but are not limited to, the use of an appropriate study design that provides a robust answer to the intended question, distinguishing between size and growth, inclusion criteria, sample size, how pregnancy is dated, measurement procedures (eg, whether or not to take replicate measurements, number of operators/assessors), quality control, and whether and how data from multiple geographical sites are combined if multiple recruitment centres are used. We discuss each of these issues in turn in the subsequent sections. First, we clarify the important distinction between charts constructed using the descriptive and prescriptive approaches.

## 2 DESCRIPTIVE VERSUS PRESCRIPTIVE APPROACHES

Growth charts are constructed using either a descriptive or prescriptive approach. The term *prescriptive approach* is used in the scientific literature to describe the process of producing biological norms or a desirable target to be achieved or aspired to at individual and population levels (so as to construct growth *standards*). Prescriptive standards show how growth should occur, independent of time and place.^17^ For human growth, these are usually based on selected populations considered to be of optimal health, for example, with adequate nutritional status and at low risk of abnormal growth. Studies that aim to develop standards use known risk factors for suboptimal growth as exclusion criteria. For example, the INTERGROWTH‐21^st^ Project had several exclusion criteria related to obstetric history, gynecological factors, sociodemographic features (eg, age, body mass index, and smoking), clinical factors (eg, blood pressure and sexually transmitted infections), and current pregnancy (eg, whether the pregnancy was a singleton with accurate pregnancy dating).^18^ A further consideration in developing a standard is the representativeness of the study population. A prescriptive approach is necessary for international relevance, as charts should be independent of place and time.

Prescriptive charts are rare, perhaps because of the distinct differences in size of children and adults observed across different populations, leading to the popular belief of inherent differences in human growth in utero. While these observable differences are generally due to epigenetics, the little evidence available has shown that only small variations in growth are explained by genetics.^19^ Until recently, it was generally accepted that observed differences in preterm, fetal, and neonatal growth were largely due to biological differences between different regions and ethnicities, resulting in a need for population‐specific charts. This concept has been challenged by evidence demonstrating similarities in the genetic makeup of different nonisolated populations worldwide^20^, ^21^ and more specifically by recent comparisons finding similarities in early and late linear fetal growth, newborn size at birth,^22^ and linear child growth^23^ in diverse populations. The concept of similarity in growth is not new. In 1974, Habicht sought to understand the effect of ethnic differences on achieving growth potential. He compared weight and height data from preschool children of different ethnic backgrounds that were presumably well nourished and found on average differences of 3% for height and about 6% for weight between preschool children of different ethnicities but comparable socioeconomic status and nutrition. In contrast, larger differences of 12% in height and 30% in weight were observed between these children that were well nourished when compared with children of similar ethnic and geographical backgrounds who lived in poor urban and rural regions.^24^ This demonstrates the importance of establishing standards that can be used to make fair comparisons between and among populations.

In contrast, the *descriptive approach* is commonly used to produce a reference chart that describes the anthropometry of a given population at a particular time and place, such as a hospital, region, or country. Descriptive reference charts are usually based on an unselected population with minimal exclusion criteria, for example, known risk factors for optimal health. Although they are used more widely, descriptive charts are only relevant to the source population. Different populations will differ in many aspects, such as rates of smoking during pregnancy, malaria, gestational diabetes, and maternal obesity, which can all affect newborn outcomes. In principle, following the descriptive approach requires separate reference charts for each subpopulation of interest.

A review of 105 charts aimed at creating newborn size reference charts revealed that only half the authors stated whether their aim was to construct a prescriptive standard or a descriptive reference. This is problematic because the two chart types have different sensitivities and specificities for detecting growth disturbances. However, even when the studies did state that their aim was to create a standard, most did not actually produce one because their study population was poorly chosen.^13^

The use of references or standards has important implications both at an individual and population level. References provide better representation of the population from which they have been constructed. In contrast, standards usually incorporate diverse populations defined loosely to represent different populations. Standards are meant to be used internationally and therefore enable direct comparisons to be made across different populations at the population level. The existence and use of different references within a country pose challenges of comparing and evaluating public health policies.^25^

## 3 STUDY DESIGN

Study design is of fundamental importance for any research study as it determines the appropriateness of the study to address the research question and helps inform the appropriate analysis of the data obtained. There are many design challenges for studies that aim to construct growth charts from fetal or neonatal measurements. Most studies are based on a cross‐sectional design^14^ that includes only one examination per fetus or newborn, whereas a longitudinal design includes measurements at more than one time. Follow‐up measures of a fetus are important for ascertaining expected growth and checking that the fetus is not growth restricted. However, as ultrasound scans are expensive, having multiple scans may not be cost effective; therefore, a trade‐off between the optimal number of scans required, their timing, and extra information gained from performing more scans would have to be evaluated. Such information would be invaluable for the planning and conducting of growth studies and is an area that requires further research.

### 3.1 Cross‐sectional, longitudinal, or mixed study designs

Assessments of how size changes over time are the most common type of analysis for fetal measurements and can be done using either a cross‐sectional or a longitudinal design. How many measurements are collected from each individual should be informed by the question being addressed. For example, size at a specific time (such as birth) can be obtained using cross‐sectional data, but ascertaining velocity requires longitudinal data. However, it is common to construct size charts from longitudinal data by simply treating them as cross‐sectional, as was done for example in the World Health Organization (WHO) Multicentre Growth Reference Study (MGRS). The nature of the data should inform the analysis methodology used. Fetal or neonatal size assessments should be related to gestational age, using some form of regression analysis. The simplest case is a pure cross‐sectional design, for example, Chitty et al took one measurement per fetus at a random time. Other longitudinal designs incorporate replicate measurements at each time point to reduce measurement error. For example, in the Fetal Growth Longitudinal Study (FGLS) of the INTERGROWTH‐21^st^ Project, ultrasound measurements of fetal size were taken in triplicate at each visit to minimise measurement error.

A mixed design incorporates both longitudinal and cross‐sectional measurements, ie, some participants are studied longitudinally and others cross‐sectionally; therefore, for any given participant, the number of measurements included may be one or greater. A mixed design can be useful for studying growth intensively in periods of rapid growth using a longitudinal design and less intensively in periods of slow growth using a cross‐sectional design. This may be an efficient, cost‐effective approach especially for multicentre studies. An example is the WHO MGRS, which combined a longitudinal study design from birth to 24 months with a cross‐sectional study of children aged 18 to 71 months.^26^ A mixed design is also likely to arise when using routine data collected from individuals requiring close monitoring who are seen more than once.

### 3.2 Size and growth

In principle, size relates to measurements at a specific time, whereas growth relates to a change in size over time. In practice, the term *growth* is widely used for both types of data and is thus sometimes used inappropriately.^27^, ^28^, ^29^ Charts derived from a single measurement of each fetus or neonate at a single time point depict size. Centile charts showing fetal size at different GAs depict the distribution of size. These size charts should not be confused with the dynamic process of growth.

True growth charts are derived from a series of anthropometric measurements made of each fetus or neonate at multiple time points.^30^, ^31^, ^32^, ^33^ Strictly speaking, only charts derived from longitudinal studies that incorporate more than one measurement per individual should be called *growth charts*. However, longitudinal studies can be used to produce both size and growth charts. Size and growth therefore refer to different information, are used in different clinical applications, and have different interpretations.

### 3.3 Who to include

The choice of an appropriate sample and target population is of great importance as comparisons and inferences applicable to the general population can be made. The target population is the population to which the chart will apply. As already discussed, this depends on whether the aim is to describe observed patterns of attained size at a specified GA (descriptive approach) or develop aspirational standards representing optimal growth. Charts produced from a subgroup of a population, such as women who are obese, would be inappropriate for making inferences about the general population as they are not representatives and lack external validity. Reference centiles for fetal size are often constructed from routinely collected data. Although retrospective analysis of such databases is a practical solution for generating a large sample size, the resulting sample will not represent optimal growth as it includes problem pregnancies, which will differ by setting. The alternative is a prospective study, in which the participant recruitment, clinical, demographic, and ultrasound data are collected with the primary objective of creating size charts and not as part of routine care provision. Data that are collected prospectively, specifically for the purpose of developing reference centiles, are therefore recommended.^31^, ^32^

### 3.4 Pregnancy dating

A reliable estimate of GA is key as it underpins clinical care and allows the expected delivery date to be estimated accurately and necessary for developing reference charts. Newborn outcomes such as preterm birth, small for GA, large for GA, and appropriate for GA are all dependent on having an accurate estimate of GA. Gestational age can be estimated early in pregnancy by using a reliable first day of the last menstrual period (LMP) alone, by using an early (9^+0^ to 13^+6^ weeks) ultrasound alone, or by combining the LMP and ultrasound. Use of the LMP is based on the assumption that pregnancy has a constant duration from the first day of the LMP, with ovulation on the 14th day.^34^ This method of dating pregnancies has been shown to be unreliable, even in women with a known menstrual history.^35^, ^36^ Caution is recommended when using the LMP alone for pregnancy dating because up to 50% of women are uncertain of their dates, have an irregular cycle, have recently stopped the oral contraceptive pill, or are lactating.^37^

Pregnancy dating by measuring the fetal crown‐rump length using an ultrasound scan is now considered an essential part of routine antenatal care. First trimester scans are recommended for confirming viability, accurately estimating GA, and determining the number of fetuses.^38^, ^39^ Crown‐rump length is reliable between 9^+0^ to 13^+6^ weeks gestation but not beyond this range.^34^ Many dating charts are now in use; however, as they have been developed using different populations, their estimated GA at any given crown‐rump length varies widely.^15^ A better approach that incorporates both ultrasound measurements of CRL and LMP with a certain level of agreement, say, 7 days, is preferred.

### 3.5 Sample size

Sample size affects centile precision and therefore must be considered when planning studies intended to develop reference charts to ensure adequate coverage of the population and any planned subgroup analysis.^26^^,^ 40 There is very limited literature on what to consider when determining the sample size of fetal growth studies.^41^, ^42^, ^43^, ^44^ In 1995, a WHO Expert Committee recommended a rule of thumb for growth studies of a sample of at least 200 individuals overall or, if relevant, for each subgroup for which separate charts will be produced.^45^ This rule of thumb is not satisfactory as it offers no guidance on the width of age groups or subgroups. With a newer statistical methodology able to cope with measurements taken at exact ages, there is no requirement for sample sizes to be based on subgroups or age groups. Cole^46^ discusses these issues in detail.

Major factors in the determination of sample size for constructing reference charts are precision and power. A sample size ought to be large enough to yield precise estimates, especially of extreme centiles (eg, the 3^rd^ and 97^th^ centiles). This of course requires a definition of *precise*, for which there is no set standard or universally agreed definition. In the case of subgroups or multicentre studies, a sufficient power may be required in order to explore ethnic‐specific (ie, site‐specific) growth. Calculating sample size is not straightforward as it depends on factors such as the study design (longitudinal, cross‐sectional, or mixed), number of repeated measurements per individual, existence of replicate measurements, and practicality (cost, time and manpower).^40^ Although statistical considerations are important, certain logistical and pragmatic issues are critical too, for example, the number of women who could be scanned at a centre in a week imposes a practical limit to what sample size can be achieved in a certain time period. The final sample size should also account for anticipated losses to follow up and for observations that may be excluded due to severe pregnancy complications or irreconcilable measurements. These factors must be considered when choosing the sample size without compromising the power of the study. A systematic review of the methodology used in published ultrasound studies for developing size or pregnancy dating charts found that only 6 of 83 published ultrasound growth or size charts included their sample size calculations in the description of their methodology.^14^, ^15^

Sample size calculations can be based on either parametric or nonparametric methods. Nonparametric methods do not make any distributional assumptions and can be implemented using simulation and bootstrap techniques.^43^, ^47^, ^48^ Regression‐based methods for sample size can also be evaluated by either nonparametric or parametric approaches, depending on the distribution of the covariate.^49^, ^50^ Methods based on regression‐based limits are commonly used in clinical chemistry studies involving normal reference ranges.^51^ The same methods can be applied in fetal and neonatal growth studies.^52^

Formulae for estimating sample size for regression‐based reference ranges were first proposed by Royston^42^ and later extended by Bellera and Hanley.^41^ In 2011, Hanley and Moodie^53^ proposed a unified approach for sample size, precision, and power calculations that considers various study designs. Later in 2016, Hanley^44^ discusses sample size considerations for the case of simple and multiple linear regressions. Regression analysis can be used to obtain reference limits that account for factors such as age, gender, and parity with corresponding confidence intervals.^54^, ^55^, ^56^, ^57^ In clinical chemistry, analytical variability is usually accounted for when developing and establishing references.^58^, ^59^ Analytical variability refers to factors likely to influence the experimental design, which includes the laboratory, day the test was taken, analyst, and instrument. In 1987, Linnet^51^ proposed that the analytical variation due to measurement error should be less than the biological variation.

Sample size calculations for growth charts based on longitudinal data are complex.^41^, ^60^ The standard errors of centiles are overestimated in longitudinal studies, as they ignore the existence of a series of measurements from each fetus.^42^, ^60^, ^61^ However, the correct use of statistical methodology, for example, mixed effects models, would avoid this issue. In general, longitudinal studies are more efficient and have greater power than cross‐sectional studies. Royston^60^ defined this efficiency as the design factor, D, which is the number of fetuses in a cross‐sectional study that would give the same precision as one fetus in a longitudinal study. He used a simulation study of ultrasound‐based biparietal diameter and compared the variance of a centile in longitudinal and equivalent cross‐sectional designs. He calculated the design factor (effect) to be ∼ 2.3.^60^ A longitudinal study thus requires approximately half to a third the sample size of a cross‐sectional study to estimate a given centile with the same precision depending on the number of measurements per fetus.

Extreme centiles exhibit large imprecision because there are few observations at the extreme ends of the distribution, whereas the median has the greatest precision. We illustrate an example based on the precision of a single centile for calculating sample size for creating reference centiles for normally distributed data. Similar approaches can be applied to non‐normal data, such as birth weight, after transformation. Other approaches also exist, for example, simulation and bootstrapping, as has been demonstrated by Harris et al,^47^ Linnet,^48^ and Jennen‐Steinmetz.^43^

### 3.6 Precision of a single centile

A reference range (also known as a reference interval or the normal range) is the range of values that encompasses the values of a physiological measurement for the large majority of healthy individuals (however defined). It forms a basis for comparison or a frame of reference for a physician or other health professional to interpret a set of test results for a particular patient or characteristic in that population. A reference interval refers to a range between two quantiles and the most commonly used interval is specified by the 2.5^th^ centile (the *lower reference limit*) and the 97.5^th^ centile (the *upper reference limit)*. It is also called the 95% reference interval as it includes 95% of measurements in a specified population. Measurement values outside a reference range are not necessarily pathologic and are not necessarily abnormal in any sense other than by an arbitrary definition. Nonetheless, values outside the reference interval are indicators of probable pathology.^62^

To estimate centiles with great precision, a large‐enough sample size, *n*, is required. Data that are conditionally normally distributed (for example, fetal size measurements) can be summarized using the mean and standard deviation (SD).^61^ Any required centile can thus be estimated from the mean and SD using the relation: μ + *z*_α_σ, where *z*_α_ is the normal equivalent deviate (z‐score) corresponding to that centile. For normally distributed unreplicated data, the standard error of the *p*_th_ centile is obtained from the standard formula for the variance of a centile as follows^63^:

$$\mathrm{SE}p=\mathrm{SD}\surd \left\{\left(1+\frac{1}{2}z_{p}^{2}\right)/n\right\},$$

where SE is the standard error, SD is the standard deviation of the measurement (which will increase with GA), *z*_*p*_ is the value of the standard normal distribution corresponding to the *p*_th_ centile, and *n* is the sample size. For example, for the 2.5^th^ or 97.5^th^ centiles, z_*p*_ = ±1.96, giving SE = 0.08 SD for a sample size of 500 and 0.03 SD for a sample size of 4000. The equation above can also be expressed as

$$\%\mathrm{SE}p=\%\mathrm{CV}\surd \left\{\left(1+\frac{1}{2}z_{p}^{2}\right)/n\right\},$$

where CV is the coefficient of variation (ie, μ/σ). For example, if the desired precision for infant length is 0.50% SE and the CV is 6%, estimating the 2.5th (or 97.5th) centiles with this precision will require an estimated minimum required sample size of 3680. Similarly, for the same precision, to estimate the 5th (or 95th) and 10th (or 90th) will require a sample size of 2800 and 2160, respectively. More extreme centiles will require a larger sample size to estimate than less extreme ones for the same precision. It is also advisable and common practice to inflate the calculated sample size by the expected percentage of attrition for the specific setting.

Further work is needed on the determination of the required sample size for growth studies. In particular, aspects of longitudinal studies have not yet been considered, such as the effect of correlations between measurements of the same individual at different ages, the number of replicates per measurement, the timing of measurements (in general, more measurements are needed in periods of more rapid growth to accurately capture the pattern of growth), and the number of observations per individual.

## 4 ROUTINELY COLLECTED DATA VERSUS RESEARCH DATA

Medical records have been used as a source of data for research since 1917, when Codman began using patient information cards to track long‐term health outcomes.^64^ Great advances in technology have contributed to a recent shift from paper‐based records to electronic medical records. Sweden and Denmark were among the first nations to make the transition to electronic record‐keeping systems that save time, money, and lives. Such comprehensive databases are gold mines of clinical information and have the potential to improve clinical practice, permit real‐time learning, and create a large evidence base for clinical care.^65^, ^66^

However, concerns have been raised about the use of patient records for research purposes. Medical records are intended for patient care and not for research. Therefore, the use of such data raises some ethical considerations as patients may not have consented to the use of their data for the purposes of research. Data are not recorded in a systematic manner, as would be the case with research.^67^ The completeness and accuracy of retrospectively collected data are thus a concern. Other difficulties include illegibility, incomplete records, and a lack of standardized documentation for ease of classification and comparability between similar settings.^68^, ^69^, ^70^ If not addressed, these concerns can bias study results and limit external validity. Villar et al^71^ showed, however, that certain obstetrical information retrieved from medical records can be reliable. In an inter‐rater agreement study of antenatal and neonatal variables collected in a large teaching obstetric unit, information routinely collected by hospital staff was compared with that collected by a specifically trained physician and social worker. They observed excellent agreement for some variables, such as maternal and newborn anthropometric measures and previous birth weight, but poor agreement for variables such as indicators of physical activity, work during pregnancy, and blood pressure measures. The poor agreement for some of the variables was hypothesized to be due to problems in how the questions were phrased, patient recall, interviewer bias, and data abstraction. The recommendation from this work was that epidemiological studies using routine data should assess, where possible, proper standardization of personnel and instruments.^71^

To be useful in research, medical data must be accurate and routinely recorded. Retrospective studies using such data would allow the investigation of rare diseases, accommodate assessment of conditions with long latency periods, and could function as pilot studies to identify weaknesses and improve study design for further prospective research.^69^

### 4.1 Quality control

One of the most important aspects of any research involving measurements is the quality of the data. Great effort is often expended on the design of a study, but little thought may be given to how the method of data collection and measurement will affect the quality of the data. In studies of fetal growth, many factors can introduce variability in ultrasound data, such as multiple sonographers and scan machines, a lack of standardized procedures when data are collected by more than one person, a lack of specific training, and failure to monitor ultrasound image quality. It has previously been argued that reference studies should be performed by a single operator to improve the repeatability of the data by avoiding interobserver error. However, as ultrasound scans in most clinical services are performed by multiple operators, interobserver variability is inevitable and should not be ignored. It is reasonable for reference studies to take interobserver variability into account when using multiple operators and to take quality assurance steps to improve the quality and consistency of measurements. Such steps include saving and independently reviewing scan images, and measuring intraobserver and interobserver variability. A formal exercise to standardize the contributing ultrasonographers should be conducted, as this improves the reproducibility of fetal biometry.^72^

In general, factors that may influence the quality, accuracy, and reproducibility of measurements include instrument error, intraobserver error, biological variation, errors in recording, bias, etc. The precision with which measurements are made also depends on the equipment used. In ultrasound studies, for example, advances in technology have improved the magnification achieved by ultrasound machines, leading to better measurements. In anthropometry, weighing equipment must have an acceptable precision. For example, the WHO MGRS and INTERGROWTH‐21^st^ studies used SECA 376 scales that were precise to 5 g for weight measurements <7.5 kg and were precise to 10 g for measurements between 7.5 and 20 kg. Measurement accuracy can further be enhanced by an explicit measurement protocol, training and standardization of the staff involved in taking measurements to avoid mistakes due to repetitiveness, assessment of image quality in the case of ultrasound images, checking for digit preference, and ongoing monitoring of adherence to the measurement protocol during data collection. For example, in the MGRS, an analysis of digit preference for one site with nine observers found that one observer tended to overestimate measurements, shown by a disproportionate frequency of the digit 0 (8.4%) versus the digit 2 (34.4%).^73^ Monitoring these elements and rectifying problems helps to ensure consistency and minimize systematic error. Alternative numerical methods of monitoring measurement processes include Bland‐Altman plots,^74^ technical errors of measurement,^75^ and cumulative sum charts.^76^, ^77^, ^78^ A review by Biau et al showed the wide applicability of quality control methods in specialties such as surgery, endoscopy, and anesthesia^79^ using a learning curve analysis to test quality.^78^, ^80^, ^81^ A detailed data quality control process that ensures good quality data is the cornerstone of any high‐quality study. For example, the INTERGROWTH‐21^st^ study collected measurements in triplicate at each instance to ensure data quality and allowed any suspect measurement to be cross‐checked with the remaining two measurements.

### 4.2 Statistical methodology

Adolphe Quetelet (1796–1874) was the first to investigate the statistical properties of anthropometry and apply the concept of the normal distribution to anthropometry data.^82^ Francis Galton (1822–1911) introduced the use of percentile scores for comparing measurements with the normal distribution using data on attained height from birth to adulthood.^83^

The main goals when constructing reference ranges is to construct centiles that change smoothly with GA, provide a good fit to the raw data, and a provide statistical model that is only as complicated as is necessary.^31^, ^32^, ^84^ Appropriate statistical approaches for constructing reference charts should be identified. Factors to consider include (a) an assessment of whether the normality assumption is reasonable, as is usually the case for fetal data conditional on GA; (b) both the mean and SD should be modeled as a function of GA in a way that accounts for the increasing variability with gestation that is typical in growth data; (c) the modeling should provide smooth centile curves; and (d) a goodness‐of‐fit assessment with graphical evaluation of the superimposed centiles should be conducted to compare the predictive model to the raw data. A review of neonatal size charts found that most of the 102 included studies described the statistical methods used, although more than half did not satisfy all of the above criteria.^13^

### 4.3 Single versus repeat measurements

In clinical practice, measurements are only made once because of clinical workload, and thus, most reference centiles are constructed using single measurements. However, in some research settings, including studies of growth, it is common to take duplicate or triplicate measurements. Repeated measurements are taken so that the mean for measurements taken in duplicate or the median for triplicate measurements^85^, ^86^ is a better (more precise) estimate of true value, to ensure data quality, and to allow estimation of within‐ and between‐variation among sonographers and anthropometrists. Using averages to develop centile charts will tend to underestimate the actual variability for single measurements. The underestimation of actual variability can be minimized by using highly experienced trained staff and strict protocols to take highly reproducible measurements.

Averaging measurements for an individual reduces measurement imprecision, resulting in tighter (or narrower) centiles than if single measurements are used. For example, in the FGLS component of the INTERGROWTH‐21^st^ project, ultrasound measurements were taken in triplicate and anthropometric measurements in duplicate at each patient visit. Statistical methods that can account for this reduced variability should be applied. Bland and Altman^74^ have dealt with a similar problem previously, though in a different context to growth data, and recommended applying a small correction to the observed variability of all of the repeated measures. In our experience, this correction does not work well for growth data as it overestimates the variability. We are unsure why it does not work well for growth data, and further research is required to explore this in detail. Other approaches such as a sensitivity analysis comparing the use of the median of triplicate or average of duplicate measurements versus the use of a single measurement selected at random ought to be considered.

## 5 SINGLE VERSUS MULTICENTRE STUDIES: HANDLING DATA FROM MULTIPLE SITES

Most studies of fetal and neonatal growth are done in a single centre. The need for a large sample size and greater generalizability leads naturally to a multicentre design, which brings additional challenges. Assessing how appropriate it is to pool data from multiple sites is challenging. The goal is to quantify differences in measurement that change over time and for multiple sites. Meta‐analysis could be used to aggregate effect sizes/metrics for a specific time point or possibly for the study period overall, but, the change in attained size is nonlinear overall and therefore cannot be easily summarized as a single unit (metric). Therefore, a meta‐analytical approach would not be suitable. Individuals within the same site tend to be more similar to each other than those from other sites. The combinability of studies in a meta‐analysis is usually judged qualitatively using the similarity of the studies, such as the similarity of the participants, interventions, and outcome variables. It is also standard practice to quantify the statistical heterogeneity of the results,^87^, ^88^, ^89^, ^90^ although this is more likely to influence the type of analysis than whether the studies can be combined.

As multicentre studies are rare in human growth studies, the combinability problem is not common. However, both the MGRS and the INTERGROWTH‐21^st^ Project were multicentre studies and so faced this problem. Statistical significance is not appropriate for judging combinability, as even unimportant differences can be statistically significant especially in very large samples. For studies like these that aimed to develop international standards, only a small amount of heterogeneity could be tolerated in the data. An additional problem is the need to judge whether the data from different sites could be combined and quantifying the differences between the sites across GA.^22^ There is no standard statistical approach for evaluating what is an acceptable level of agreement. It is recommended to set in advance the criterion for judging whether the differences between the centile curves from each site are acceptable before conducting the analysis.

The INTERGROWTH‐21^st^ Project used the same criterion as the MGRS where the impact of the consistency and magnitude of differences in each site compared with all sites was judged according to Cohen^91^ with differences of 0.5 SD considered to be medium (an ideological criterion rather than a statistical criterion). This criterion is also widely used in the assessment and evaluation of changes in health‐related quality‐of‐life measures and patient‐reported outcomes.^92^ For the INTERGROWTH‐21^st^ Project and WHO‐MGRS, it was decided before the analysis that a difference of 0.5 SD or greater between the centile curves from a site and the combined data from all of the sites at any GA would indicate that the data from that site were too different to be pooled.^23^, ^84^ If none of the data from all eight sites exceeded this criterion in all of the analyses, then all of the data would be pooled and used to construct international standards. The INTERGROWTH‐21^st^ Project evaluated the impact on fitted centiles of excluding each site's data one at a time when compared with a pooled analysis of all sites. This was useful in judging if one site was hugely different from the rest. It is recommended that multicentre studies should quantify and evaluate the differences between their sites using prespecified criteria, as was done in the INTERGROWTH‐21^st^ Project^22^ and WHO.^23^

### 5.1 Other considerations: Reporting and presentation of results

Altman and Chitty^31^ and Royston and Altman^33^ discussed and recommended appropriate ways of reporting and presenting studies to develop reference charts. A table of included observations should be reported that shows how many individuals or women were recruited in each GA window (eg, each week of pregnancy) with the mean, SD, and associated sample size for each measurement at each completed GA. Another table should show selected fitted centile values (eg, the 10th, 50th, and 90th), and regression equations should be provided that enable the calculation of any desired centiles and z‐scores.

The systematic review of 83 fetal size studies by Ioannou et al^14^ found variable reporting of results in the vast majority of the publications included. Most of the included studies (82%) reported tables with median values and 78% included selected centiles. Sixty percent of the included studies reported equations for the mean or median, but only 39% reported equations for the SD, either as a fixed number or as a function of gestation. Equations enable calculation of desired centiles and are relevant for comparisons with other studies using Z‐scores (or parameters that allow them to be computed).

A goodness‐of‐fit assessment, with graphical evaluation of the superimposed centiles, is essential for assessing a predictive model. A smooth change in the mean superimposed onto the raw data should be reported to allow model assessment. The fitted centiles alone without superimposing raw measurement data do not allow judgments of model fit to be made. A review of 102 neonatal size charts found that a quarter of the studies reported only raw centiles instead of smoothed centiles.^13^ In other cases, the regression model used to smooth the centiles was poorly described or not appropriate for the purpose. About 25% of the studies included in the review did not report any centiles and z‐scores could be computed for only 16% of these studies.

## 6 SUMMARY

Altman and Chitty^31^ discussed some of the considerations for the design and methodology of studies of fetal size with the aim of improving the quality of future studies. Royston and Altman^33^ discussed longitudinal studies of fetal size for similar reasons. Ioannou et al^14^ reported a positive correlation between quality scores and year of publication, showing that the methodological quality of fetal size is steadily improving thanks to efforts such as these. However, the INTERGROWTH‐21^st^ systematic reviews of pregnancy dating and fetal and newborn charts showed that many studies of fetal size are still conducted poorly.^13^, ^14^, ^15^ For example, the INTERGROWTH‐21^st^ review of pregnancy dating charts identified 29 studies with the main aim of constructing charts to predict GA from crown‐rump length.^15^ The four studies with the lowest risk of bias showed the smallest variation in their predicted GA when compared. Another review of the methodology used in ultrasound studies aiming to create charts of fetal size identified 83 studies. The highest potential for bias was found in the inclusion and exclusion criteria, ultrasound quality control measures, and not having a statement on sample size determination. A review of 105 newborn charts revealed shortcomings in anthropometric evaluation, GA estimation, assessment of outliers, and chart presentation.^13^ Many of these charts are in clinical use today and directly affect the identification of at‐risk newborns who require treatment and nutritional strategies.

In summary, we have reiterated some of the concepts identified as important for growth studies, focusing on considerations and concepts related to study design. We have provided details and practical examples based on our experiences from the INTERGROWTH‐21^st^ Project. We hope that the design and methodological considerations we have discussed here will be useful for researchers in improving the design of studies aiming to construct reference or standard charts or in the assessment of such studies.

## COMPETING INTERESTS

The authors declare that they have no competing interests.

## AUTHORS' CONTRIBUTIONS

EOO wrote the manuscript with contributions from DGA. DGA read and approved earlier versions of the manuscript.

## AUTHORS' INFORMATION

EOO is a Senior Medical Statistician and DGA is a Professor of Statistics in Medicine.
